# Next-generation proteasome inhibitor MLN9708 sensitizes breast cancer cells to doxorubicin-induced apoptosis

**DOI:** 10.1038/srep26456

**Published:** 2016-05-24

**Authors:** Hao Wang, Yang Yu, Zheng Jiang, Wen-Ming Cao, Zhenyu Wang, Jun Dou, Yanling Zhao, Yunfu Cui, Hong Zhang

**Affiliations:** 1 Department of Hepatopancreatobiliary Surgery, Second Affiliated Hospital of Harbin Medical University, Harbin, Heilongjiang 150086, China; 2 Department of Pathology, The University of Texas MD Anderson Cancer Center, Houston, Texas 77030, USA; 3 Laboratory of Medical Genetics, Harbin Medical University, Harbin, Heilongjiang 150081, China; 4 Department of Colorectal Surgery, Cancer Hospital, Chinese Academy of Medical Sciences, Peking Union Medical College, Beijing 100021, China; 5 Department of Medical Oncology, Zhejiang Cancer Hospital, Hangzhou, Zhejiang 310022, China; 6 Department of Breast Surgery, Second Hospital of Jilin University, Changchun, Jilin 130041, China; 7 Xinjiang Key Laboratory of Plant Resources and Natural Products Chemistry, Xinjiang Technical Institute of Physics and Chemistry, Chinese Academy of Sciences, Urumqi, Xinjiang 830011, China; 8 Department of Molecular and Translational Pathology, The University of Texas MD Anderson Cancer Center, Houston, Texas 77030, USA

## Abstract

Doxorubicin (Dox), one of the most effective chemotherapy drug for cancer treatment, is limited by its severe side effects and chemoresistance. Dox induces DNA damage and leads to significant proteomic changes in the cancer cells, which makes the ubiquitin-proteasome system a potential target to enhance the efficacy of Dox therapy. The unsuccessful clinical trials of proteasome inhibitor PS-341 (bortezomib) in solid tumors led to the invention of MLN9708 (ixazomib), an orally bioavailable next-generation proteasome inhibitor with improved pharmacokinetic and pharmacodynamic features. In this preclinical study, we used eight human breast cancer cell lines, which represent the major molecular subtypes of breast cancer, to validate the cytotoxic effects of MLN9708, alone and in combination with Dox. We found that MLN9708 had cytotoxic effects, induced autophagy and MKP-1 expression, and enhanced Dox-induced apoptosis in these cell lines. MLN9708 also enhanced Dox-induced JNK and p38 phosphorylation and inhibited Dox-induced IκBα degradation. Our *in vitro* results suggest that MLN9708 has antitumor effects in breast cancer and can sensitize breast cancer cells to Dox treatment. This promising combination may be an effective and feasible therapeutic option for treating breast cancer and warrants clinical validation.

Doxorubicin (Dox)-containing adjuvant cytotoxic chemotherapy is considered the standard of care for breast cancer, according to the 2015 National Comprehensive Cancer Network guidelines^1^,^2^. As an anthracycline antibiotic, Dox works in all phases of the cell cycle. This topoisomerase II poisoning regimen has been widely used in anticancer therapies. Dox interferes with DNA synthesis, induces DNA damage, produces reactive oxygen species, and destroys membrane structure in the treated cells^3^,^4^,^5^,^6^. However, severe side effects, such as life-threatening cardiotoxicity, strictly limit Dox dosage^7^. Thus, other reagents or small molecules that can enhance the therapeutic effects of Dox are highly desirable and are being actively assessed in the laboratory and in the clinical setting^8^.

Studies show that the cytotoxic effects of Dox cause significant ubiquitin-proteasome system-mediated proteomic changes which are vital for cell survival in the treated cells^9^,^10^. The proteasome (multicatalytic proteinase complexes in eukaryotic cells) is responsible for the regulation and degradation of most intracellular proteins, some of which mediate cell-cycle progression and apoptosis, such as cyclins, caspases, and nuclear factor of κB (NF-κB)^11^. The NF-κB family of transcription factors plays critical roles in controlling inflammation, the immune response, and anti-apoptotic responses^12^,^13^. Inhibiting the activation of NF-κB promotes cell death, which has become a promising anticancer strategy^14^. Several studies have verified that inhibiting the proteasome can suppress the degradation of nuclear factor of kappa light polypeptide gene enhancer in B-cells inhibitor (IκB), which inhibits NF-κB nuclear translocation and activation^15^,^16^. The proteasome system also plays an important role in the regulation of DNA damage response and is highly involved in the DNA repair process^17^,^18^. Additionally, because of their genetic instability and rapid proliferation, cancer cells tend to be more dependent on the proteasome than normal cells for the removal of aberrant intracellular proteins^10^,^11^. Therefore, functional inhibition of proteasome activity may disturb numerous cellular activities and lead to cancer cell death.

The first generation proteasome inhibitor PS-341 (bortezomib) has been approved by the United States Food and Drug Administration (FDA) for the treatment of many hematological malignancies. However, the results from clinical trials indicate that PS-341 and PS-341–containing therapies are not effective for the treatment of solid tumors including breast cancer due to the inability of PS-341 to penetrate into tumors and achieve therapeutically relevant concentrations in tumor^19^,^20^,^21^,^22^.

MLN9708 (ixazomib), the next-generation proteasome inhibitor, has been shown to have potent anticancer activity in both hematologic and solid tumor xenograft models with better pharmacokinetic and pharmacodynamic features than PS-341^23^. MLN9708 can be orally administrated, which is more convenient for clinical practice. Accumulating evidence indicates that MLN9708 could be a possible therapy for the treatment of solid tumors including breast cancer^24^,^25^.

Until now, the potential therapeutic effects of MLN9708 on breast cancer remain unknown^23^. In this preclinical study, by using a panel of breast cancer cell lines including T47D, MCF7, MDA-MB-361, SK-BR-3, HCC1954, MDA-MB-468, MDA-MB-231, and BT-549 (representing ER/PR+/−, HER2+, or triple negative, respectively) (Table 1)^26^,^27^,^28^, we examined the cytotoxic effects of MLN9708 and whether MLN9708 could sensitize breast cancer cells to Dox-induced apoptosis.

## Results

### MLN9708 suppresses the proliferation of breast cancer cells

**Figure 1:**
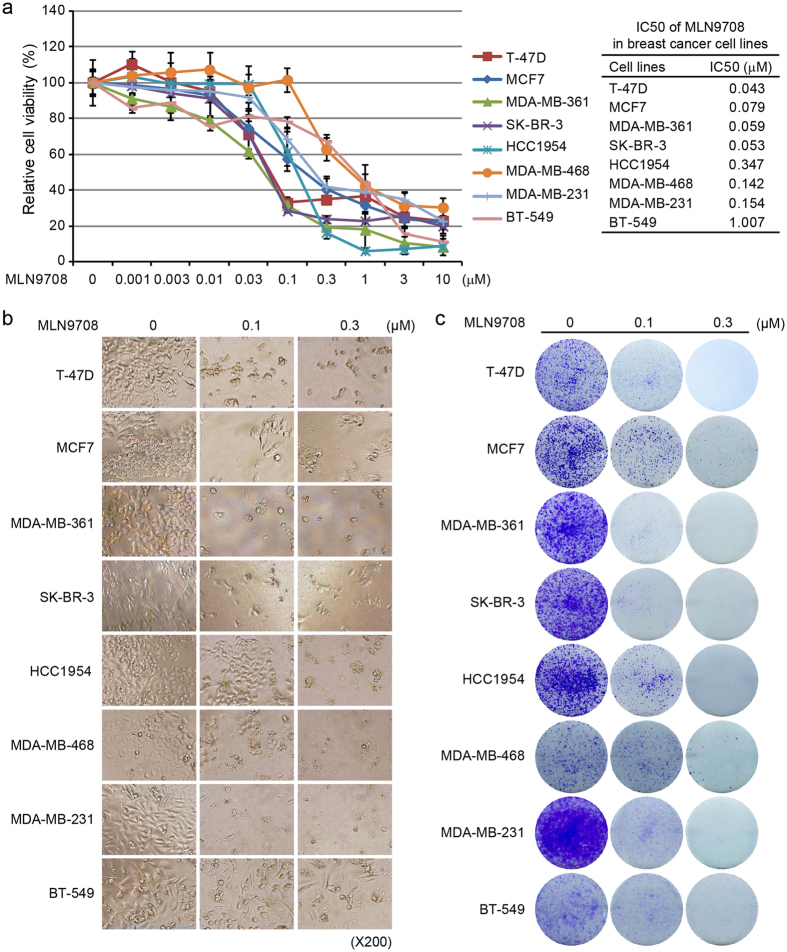
MLN9708 shows cytotoxic effect in breast cancer cells. (**a**) Cytotoxic effect of MLN9708 on breast cancer cells. Eight human breast cancer cell lines (T-47D, MCF7, MDA-MB-361, SK-BR-3, HCC1954, MDA-MB-468, MDA-MB-231, and BT-549) were incubated with medium alone or were treated with MLN9708 (0.001 μM, 0.003 μM, 0.01 μM, 0.03 μM, 0.1 μM, 0.3 μM, 1 μM, 3 μM, or 10 μM) for 72 h and then were subjected to a Cell Counting Kit-8 (CCK-8) assay. The absorbance of each well was measured at 450 nm, and the cell viability curve was plotted. Data were represented as means ± standard deviations (SD). Median inhibitory concentration (IC_50_) values of MLN9708 in breast cancer cell lines were listed. (**b**) Photographs of treated cells (magnification, ×200). (**c**) Colony formation of breast cancer cells treated with MLN9708. Cells were seeded in 6-well plates (5 × 10^3^ per well), were incubated with medium alone or with MLN9708 (0.1 μM or 0.3 μM) for 72 h, and then were cultured in drug-free medium for 2 weeks. The cell colonies were fixed, were stained with crystal violet, and were photographed.

To assess the antitumor effect of MLN9708 on breast cancer cells, we selected eight breast cancer cell lines (T-47D, MCF7, MDA-MB-361, SK-BR-3, HCC1954, MDA-MB-468, MDA-MB-231, and BT-549), which represent the major molecular subtypes of breast cancer (Table 1)^26^,^27^,^28^. All cells were incubated with medium alone (control) or were treated with MLN9708 at the indicated concentrations (0.001 μM–10 μM) for 72 h and were subjected to a Cell Counting Kit-8 (CCK-8) assay. MLN9708 reduced the viability of all types of breast cancer cells in a dose-dependent manner (Fig. 1a). The cytotoxic effect of MLN9708 was confirmed by morphological images of the cells after treatment for 72 h (Fig. 1b). Since the median inhibitory concentration (IC_50_) values of MLN9708 were around the doses of 0.1 μM and 0.3 μM within all cell lines, we only show data from samples treated with these two doses.

To validate the effect of MLN9708 on cell growth, we performed cell colony formation assays. The cells were incubated with medium alone or were treated with MLN9708 at concentrations of 0.1 μM or 0.3 μM for 72 h and then were cultured in drug-free medium for 2 weeks. MLN9708-treated cells had remarkably less proliferation potenital than the control groups (Fig. 1c). These data indicate that MLN9708 had a potent inhibitory effect on breast cancer proliferation, regardless of molecular subtype.

### MLN9708 impairs the anchorage-independent growth of breast cancer cells

**Figure 2:**
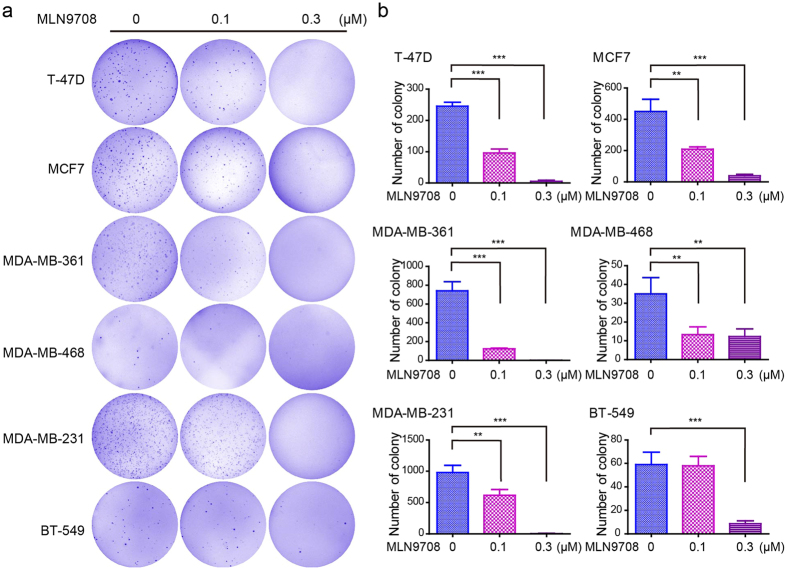
MLN9708 suppresses anchorage-independent growth in breast cancer cells. (**a**) Cell anchorage-independent growth ability was assessed by soft agar assay. Six breast cancer cell lines (T-47D, MCF7, MDA-MB-361, MDA-MB-468, MDA-MB-231, and BT-549) were incubated with MLN9708 (0 μM, 0.1 μM, or 0.3 μM) in soft agar plates for 3 weeks. Cells were then stained with crystal violet and were photographed. (**b**) The colonies were counted, and the data were plotted. Data were represented as means ± standard deviations (SD). *P < 0.05, **P < 0.01, and ***P < 0.001 (analysis of variance and Dunnett multiple comparison post-test).

Cancer cells can grow colonies by forming a three-dimensional sphere in soft agar, a process called anchorage-independent growth. To assess whether MLN9708 could inhibit the anchorage-independent growth of breast cancer cells, we performed soft agar assays. Breast cancer cells (T-47D, MCF7, MDA-MB-361, SK-BR-3, HCC1954, MDA-MB-468, MDA-MB-231, and BT-549) were cultured with MLN9708 (0.1 μM or 0.3 μM) for 3 weeks. Then the visible colonies were fixed and stained. Untreated cells were used as controls. Because HCC1954 and SK-BR-3 cells did not form visible colonies in soft agar assay, data from these cell lines are not shown. The MLN9708-treated groups had fewer colonies than the control groups in the tested cell lines (Fig. 2a). MLN9708 significantly inhibited colony formation in a dose-sensitive manner in the tested cells (Fig. 2b).

### MLN9708 induces apoptosis in breast cancer cells

**Figure 3:**
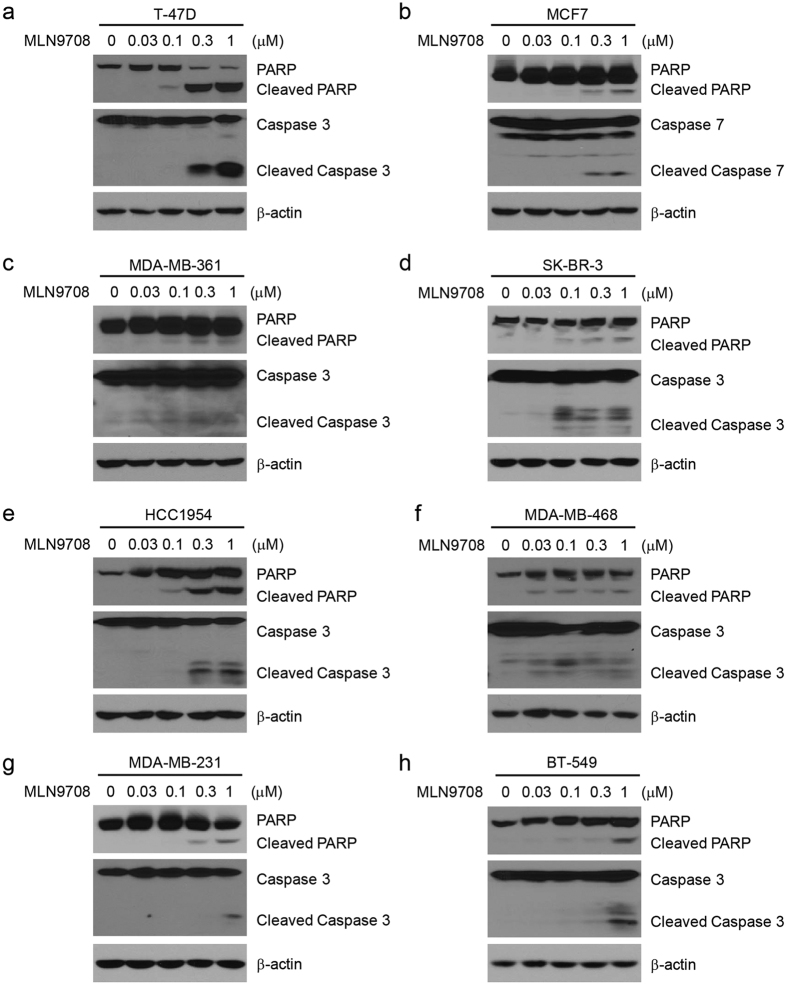
MLN9708 induces apoptosis in breast cancer cells. (**a–h**) Breast cancer cells (T-47D, MCF7, MDA-MB-361, SK-BR-3, HCC1954, MDA-MB-468, MDA-MB-231, and BT-549) were treated with MLN9708 (0.03 μM, 0.1 μM, 0.3 μM, or 1 μM) for 24 h. Untreated cells were used as controls. Whole-cell lysates were subjected to SDS-PAGE and were immunoblotted with antibodies against PARP and Caspase 3 (or Caspase 7) to detect apoptosis. β-actin was used as a loading control.

Studies have reported that MLN9708 induces apoptosis in many types of tumors, including multiple myeloma and lymphoma^29^,^30^. To determine whether MLN9708 induces apoptosis in human breast cancer cells, we treated the eight breast cancer cell lines with MLN9708 at different concentrations (0 μM, 0.03 μM, 0.1 μM, 0.3 μM, or 1 μM) for 24 h. Cells were then harvested and subjected to immunoblotting assays. Since MCF7 cells are Caspase 3 deficient, we measured Caspase 7 levels for this cell line. We found that MLN9708 induced poly (ADP-ribose) polymerase (PARP) and Caspase 3 (or Caspase 7) cleavage in the tested cell lines in a dose-dependent manner (Fig. 3a–h). These results suggest that MLN9708 alone triggered apoptosis in breast cancer cells.

### MLN9708 induces autophagy activation in breast cancer cells

**Figure 4:**
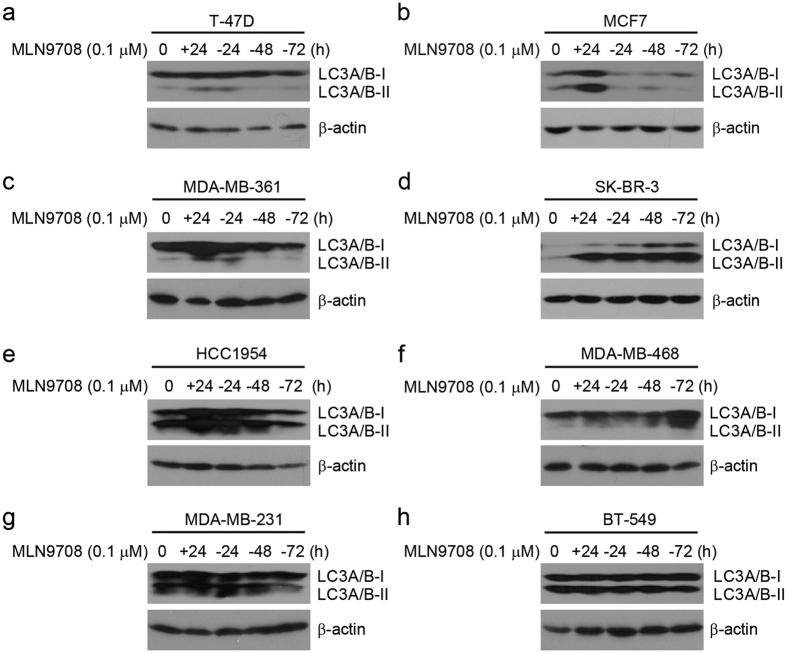
MLN9708 induces autophagy in breast cancer cells. (**a–h**) Breast cancer cells (T-47D, MCF7, MDA-MB-361, SK-BR-3, HCC1954, MDA-MB-468, MDA-MB-231, and BT-549) were treated with MLN9708 (0.1 μM) for 24 h and then were cultured with drug-free medium for 24 h, 48 h, or 72 h. Then whole-cell lysates were subjected to SDS-PAGE and were immunoblotted with antibodies against LC3A/B-I/II to detect autophagy. β-actin was used as a loading control.

Because studies have proposed induction of autophagy as a resistance mechanism to proteasome inhibitor treatment in breast cancer cells^31^,^32^, we performed immunoblotting analysis to determine how MLN9708 treatment affects the autophagy machinery. Cells were treated with MLN9708 (0.1 μM) for 24 h and then were incubated with drug-free medium for 24 h, 48 h, or 72 h. The results show that MLN9708 induced autophagy marker LC3A/B-I/II expression in the cell lines tested (Fig. 4a–h). The response of individual cell lines to the addition or withdrawal of MLN9708 varied (Fig. 4a–h). The results suggest that autophagy machinery plays an important role in proteasome inhibitor treatment resistance in breast cancer cells.

### MLN9708 induces MKP-1 expression in breast cancer cells

**Figure 5:**
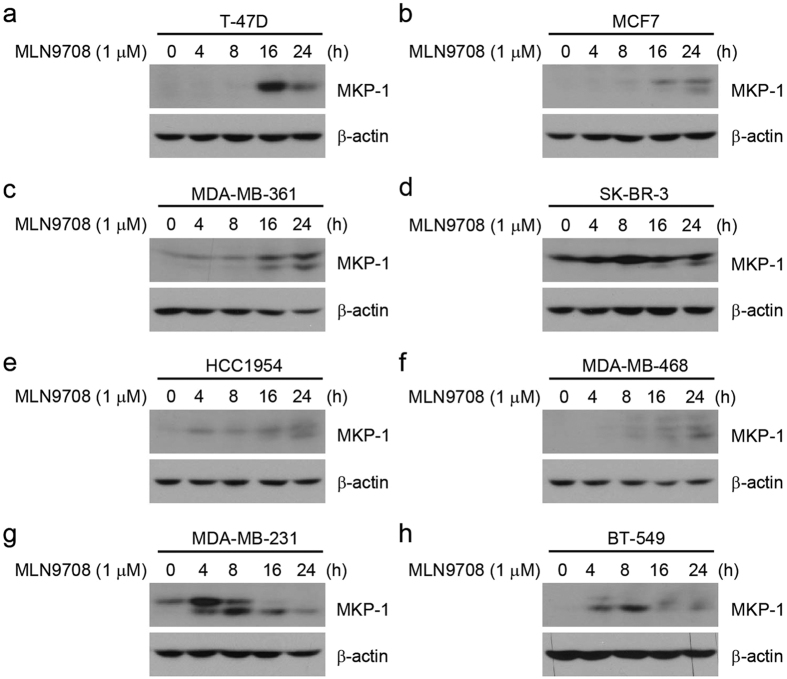
MLN9708 induces MKP-1 expression in breast cancer cells. (**a–h**) Breast cancer cells (T-47D, MCF7, MDA-MB-361, SK-BR-3, HCC1954, MDA-MB-468, MDA-MB-231, and BT-549) were treated with MLN9708 (1 μM) for 4 h, 8 h, 16 h, or 24 h. Untreated cells were used as controls. Then whole-cell lysates were subjected to SDS-PAGE and were immunoblotted with antibodies against MKP-1. β-actin was used as a loading control.

Because several studies have suggested that the buildup of mitogen-activated protein kinase phosphatase-1 (MKP-1) may account for the chemoresistance of cancer cells to proteasome inhibitors^33^,^34^,^35^, we assessed the effect of MLN9708 on the induction of MKP-1 expression in the eight breast cancer cell lines. Cells were cultured in medium alone or with MLN9708 (1 μM) for 4 h, 8 h, 16 h, or 24 h. We found that MLN9708 induced MKP-1 expression (Fig. 5a–h).

### MLN9708 enhances the cytotoxic effect of Dox on breast cancer cells

**Figure 6:**
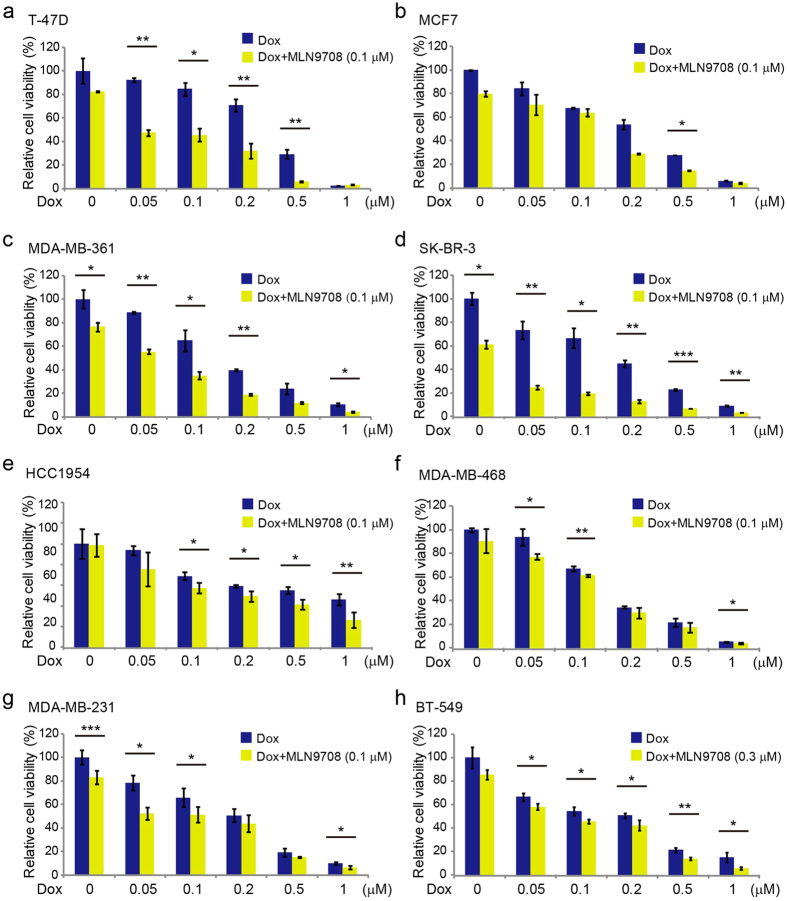
MLN9708 enhances the cytotoxic effects of doxorubicin (Dox) in breast cancer cells. (**a–h**) Breast cancer cells (T-47D, MCF7, MDA-MB-361, SK-BR-3, HCC1954, MDA-MB-468, MDA-MB-231, and BT-549) were treated with Dox at the indicated concentrations with or without MLN9708 (0.1 μM [0.3 μM for BT-549 cells]) for 48 h. Cell viability was then measured by Cell Counting Kit-8 (CCK-8) assay. Data were represented as means ± standard deviations (SD). *P < 0.05, **P < 0.01, and ***P < 0.001 (*t*-test).

To determine whether MLN9708 enhances the cytotoxic effects of Dox in breast cancer cells, we cultured the cells in increasing concentrations of Dox alone or in combination with MLN9708 (0.1 μM [0.3 μM for BT-549 cells]) for 48 h and assessed the cell viability using CCK-8 assay. Cells in the combination groups had much lower viability than cells treated with Dox alone (Fig. 6a–h). These findings imply that MLN9708 sensitized breast cancer cells to Dox-mediated cytotoxicity.

### MLN9708 enhances Dox-induced apoptosis in breast cancer cells

**Figure 7:**
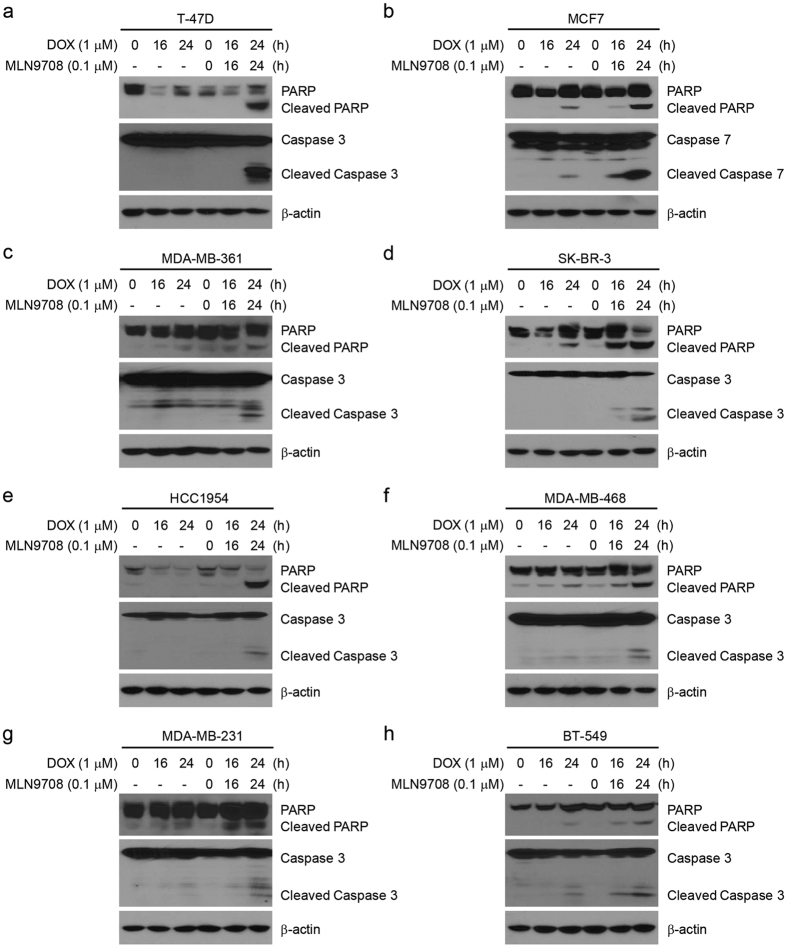
MLN9708 enhances doxorubicin (Dox)-induced apoptosis in breast cancer cells. (**a–h**) Breast cancer cells (T-47D, MCF7, MDA-MB-361, SK-BR-3, HCC1954, MDA-MB-468, MDA-MB-231, and BT-549) were treated with Dox (1 μM) alone or in combination with MLN9708 (0.1 μM) for 16 h or 24 h. Untreated cells were used as controls. Then whole-cell lysates were subjected to SDS-PAGE and were immunoblotted with antibodies against PARP and Caspase 3 (or Caspase 7) to detect apoptosis. β-actin was used as a loading control.

To explore whether MLN9708 enhances Dox-induced cell apoptosis in breast cancer cells, we treated the cells with Dox alone (1 μM) or in combination with MLN9708 (0.1 μM) for 16 h or 24 h. Untreated cells were used as controls. Immunoblotting results demonstrated that MLN9708 enhanced Dox-induced PARP and Caspase 3 (or Caspase 7) cleavage in all subtypes of breast cancer cells tested (Fig. 7a–h). These findings indicate that MLN9708 intensified Dox-induced apoptosis in breast cancer cells.

### MLN9708 inhibits Dox-induced IκBα degradation

**Figure 8:**
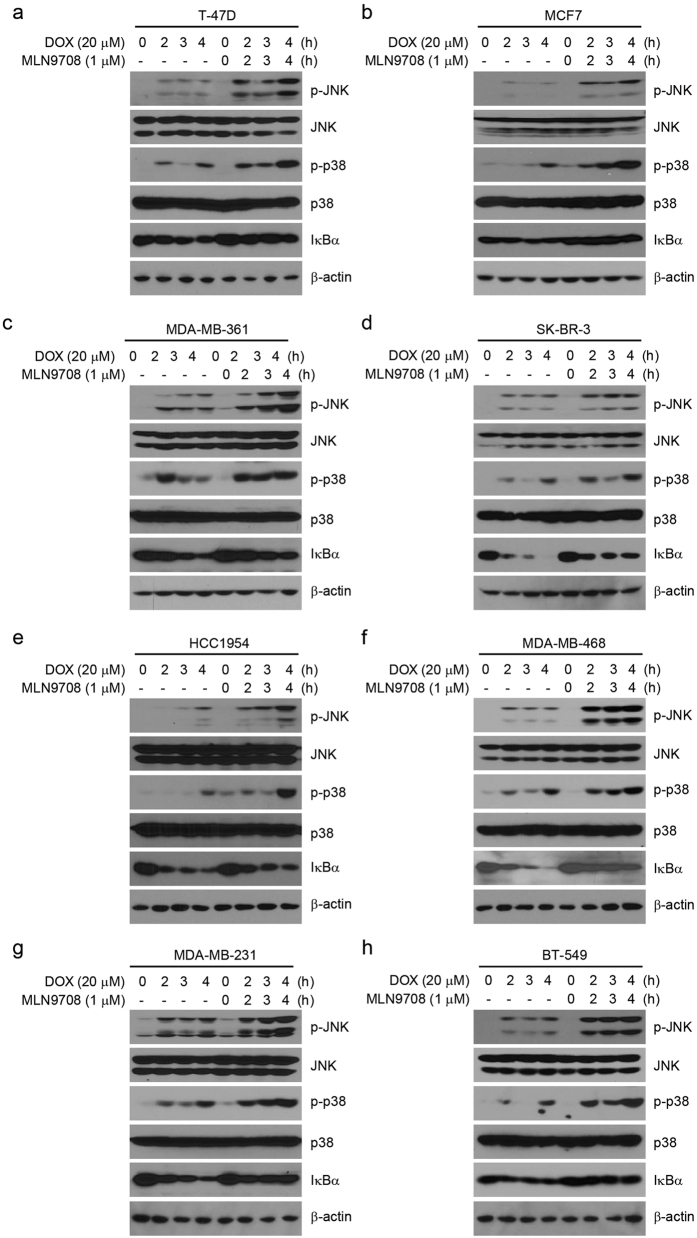
MLN9708 inhibits doxorubicin (Dox)-induced IκBα degradation in breast cancer cells. (**a–h**) Breast cancer cells (T-47D, MCF7, MDA-MB-361, SK-BR-3, HCC1954, MDA-MB-468, MDA-MB-231, and BT-549) were treated with Dox (20 μM) alone or in combination with MLN9708 (1 μM) for 2 h, 3 h, or 4 h. Untreated cells were used as controls. Then whole-cell lysates were subjected to SDS-PAGE and were immunoblotted with antibodies against p-JNK, JNK, p-p38, p38, and IκBα. β-actin was used as a loading control.

Both the NF-κB and mitogen-activated protein kinases (MAPK) pathways are important for regulating cell proliferation and survival^36^,^37^,^38^. Studies have shown that Dox induces NF-κB and MAPK activation and that proteasome inhibitors inhibit the activation of NF-κB^39^,^40^,^41^. To clarify this interaction, we assessed the effects of the combination of MLN9708 with Dox on the activity of NF-κB and MAPK in the eight breast cancer cell lines. Cells were cultured with Dox alone or in combination with MLN9708 (1 μM) for 2 h, 3 h, or 4 h. Untreated cells were used as controls. MLN9708 enhanced Dox-induced c-Jun N-terminal kinase (JNK) and p38 phosphorylation but suppressed Dox-induced IκBα degradation (Fig. 8a–h).

## Discussion

Studies have revealed that inhibiting the proteasome activity disturbs the regulation and degradation of most intracellular proteins, including cyclins, cell-cycle–dependent kinase inhibitors, and proapoptotic proteins^18^. Moreover, the buildup of incorrectly folded proteins and proteins with a short half-life (mostly with regulatory functions) leads to cell destruction^16^. In this study, we examined the cytotoxic effects of MLN9708, the next-generation proteasome inhibitor, in breast cancer cells. We found that MLN9708 exhibited general antitumor activity in eight breast cancer cell lines.

Breast cancer is a heterogeneous group of diseases and is classified into several major subtypes, including the luminal type that expresses estrogen receptor, the HER2–enriched type, and basal-like breast cancers that comprise most triple-negative tumors^26^,^27^. Each subtype may have a specific response to different drugs. We investigated the general effects of MLN9708 on a panel of eight breast cancer cell lines, which represent the major subtypes of breast cancer (ER/PR+/−, HER2+, or triple-negative). We found that MLN9708 was cytotoxic in breast cancer cells in a dose-dependent manner (Fig. 1) but with varying efficacy. The IC_50_ values of MLN9708 in the luminal subtypes (T-47D, MCF7, and MDA-MB-361) were less than 0.1 μM, indicating that luminal (ER+) cancer cells are sensitive to MLN9708. This observation suggests that MLN9708 could be a complementary single agent for current endocrine therapy with reasonable toxicity. Triple-negative cells (MDA-MB-468, MDA-MB-231, and BT-549) had higher IC_50_ values and varied more dramatically than the other two major subtypes, indicating that MLN9708 would have less efficacy as a single-agent regimen for these patients than for those with other subtypes (Fig. 1). The IC_50_ values of HER2 + cells (SK-BR-3 and HCC1954) were intermediate (ranging from 0.05 μM to 0.5 μM), suggesting that patients with HER2 + breast cancer might benefit from MLN9708-containing therapy. IC_50_ values are dependent on the conditions under which they are measured, such as the number of cells in the sample and treatment duration. Our analysis roughly reflects the sensitivity of cell lines to MLN9708 treatment at 5,000 cells per well for 72 h.

Tumor heterogeneity characterizes cancer cells that display distinguishable phenotypic features, including cellular morphology, gene expression profile, metabolism, motility, and proliferation potential^42^,^43^. Although the IC_50_ values of MLN9708 on T-47D and BT-549 cells were different, the colony formation of these two cell lines treated with MLN9708 (0.1 μM or 0.3 μM) was similar (Fig. 1). We also found that MCF7, MDA-MB-361, and SK-BR-3 cells had similar MLN9708 IC_50_ values (Fig. 1a), but their colony formation results varied, especially when treated with 0.1 μM of MLN9708 (Fig. 1c). These differences might be due to variation of the diversity of cancer cell lines in cell size, morphology, doubling time, etc. The numbers of living cells from different cell lines differed after treatment with MLN9708 for 72 h. In addition, two weeks later with drug-free medium culture, the proliferation rate of the cell lines also varied. All of these factors contributed to the variations of the colony formation potential of the breast cancer cells. Regardless, MLN9708 inhibited the proliferation, colony formation, and anchorage-independent growth of breast cancer cells in a dose-dependent manner (Figs 1 and 2).

Dox treatment interferes with many intracellular biological reactions and simultaneously generates countless molecules, such as unfolded or misfolded proteins, peptides, and lipoproteins. To maintain normal cellular function and viability, the proteasome will degrade most of this cellular waste after ubiquitination^10^,^11^,^15^. When the degradation or removal process of this cellular waste is prolonged, cells die. As expected, several studies have demonstrated that proteasome inhibition led to the inhibition of the general DNA damage response and other vital processes^17^,^18^,^44^,^45^,^46^. These findings theoretically support the ability of proteasome inhibitors to sensitize cancer cells to other cytotoxic agents. Although the mechanism of the effects of two cytotoxic agents on cancer cells is difficult to be determined owing to the complexity of the cellular response, it is reasonable and feasible to use the end results, i.e., the killing of cancer cells, as an indicator to identify effective treatment regimens.

As shown in our studies, MLN9708 enhanced Dox-induced cytotoxicity (Fig. 6) and Dox-induced apoptosis in breast cancer cells that represent the major subtypes of breast cancer (Fig. 7). In addition to sensitizing breast cancer cells to Dox, MLN9708 also might lower the effective dose of Dox and achieve similar or better therapeutic effects with fewer side effects.

JNK and p38 are the master mediators for chemotherapeutic drug-induced cell death^47^,^48^. When we used JNK and p38 to assess cellular stress leading to apoptosis^40^, we found that MLN9708 enhanced Dox-induced phosphorylation of JNK and p38 (Fig. 8). The activation of NF-κB plays a pivotal role in promoting oncogenesis and resistance to chemotherapy^49^,^50^,^51^,^52^,^53^. NF-κB was reported to be activated in response to genotoxic stresses, such as Dox or VP16 treatment, and inhibition of NF-κB activation enhanced the effect of cytotoxic agents-induced cell death^39^,^49^,^50^. In support of these previous findings, we found that MLN9708 inhibited Dox-induced IκBα degradation (Fig. 8), which might block the translocation of NF-κB to the nucleus and the activation of NF-κB-dependent anti-apoptotic gene expression.

Although our study demonstrated that MLN9708 has potent cytotoxicity, potential mechanisms of chemoresistance to MLN9708 should be considered. Activation of autophagy has been proposed as a resistance mechanism to proteasome inhibitor MG132 and PS-341 treatment in breast cancer cells^31^,^32^, which suggests that combination with an autophagy inhibitor would be beneficial. We also observed that MLN9708 induced autophagy (Fig. 4). Compared with the other cell lines, cell lines with higher MLN9708 IC_50_ values (HCC1954, MDA-MB-231, and BT-549) had a high level of basal autophagy, which supports the findings of Gavilán *et al*.^31^. Other studies have suggested that the buildup of MKP-1 may account for chemoresistance of cancer cells to proteasome inhibitors^33^,^34^,^35^. Supporting these studies, we observed that MLN9708 induced buildup of MKP-1 (Fig. 5), which suggests that combining MLN9708 with appropriate regimens against chemoresistance could improve outcomes in cancer therapy.

**Figure 9:**
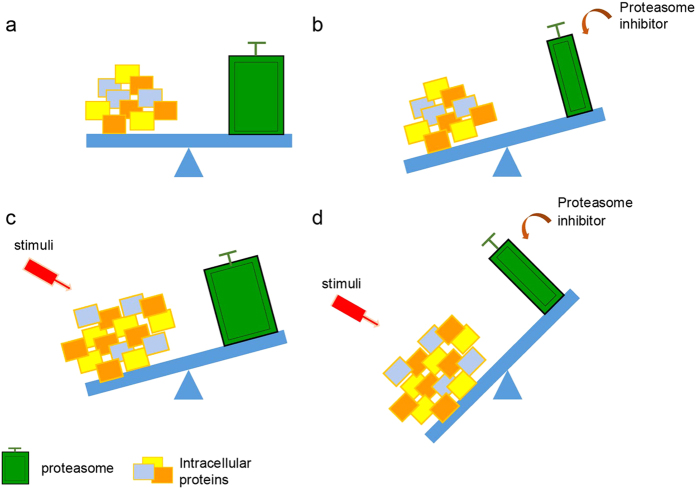
Working model shows cytotoxic effects of MLN9708 in breast cancer. (**a**) The balance between intracellular proteins and the functional proteasome in living cells. (**b**) Loss of balance in cells treated with proteasome inhibitor MLN9708. (**c**) Doxorubicin (Dox)-induced changes of intracellular proteins challenge the working capacity of the proteasome. (**d**) Combination of MLN9708 with Dox further stresses the cells and causes apoptosis.

Based on the previous studies with proteasome inhibitors and Raymond’s proteotoxic crisis hypothesis^10^,^18^,^54^, together with our observations in this study, we propose a working model regarding the role of proteasome inhibitors in cancer cells (Fig. 9). In this model, the balanced relationship between intracellular proteins and the functional proteasome in living cells is called proteostasis (Fig. 9a). When the cells are treated with a proteasome inhibitor, proteostasis is difficult to be maintained due to the impairment of the proteasome, which leads to increased accumulation of intracellular proteins and creates a crisis of proteostasis (Fig. 9b). DNA damage-inducing stimuli or other treatments greatly change the qualities, quantities, or distribution of intracellular proteins, which challenges the working capacity of the proteasome (Fig. 9c). The combination of a proteasome inhibitor with conventional cytotoxic drugs further challenges the processing ability of the proteasome, which ultimately leads to cell death (Fig. 9d). Our working model simplifies the possible therapeutic mechanisms of combining proteasome inhibitors with other conventional cytotoxic agents.

In conclusion, our findings demonstrate for the first time that the second-generation proteasome inhibitor MLN9708 is cytotoxic to various breast cancer cells (ER/PR+/−, HER2+, or triple-negative) and can induce cell death. MLN9708 enhances the anticancer effects of Dox by upregulating Dox-induced activation of JNK and p38 and inhibiting Dox-induced NF-κB activation. Our findings suggest that this combination strategy might be beneficial in treating breast cancer and warrant further investigation and clinical validation.

## Materials and Methods

### Cell lines and cell culture

Human breast cancer cell lines T-47D, MCF7, MDA-MB-361, SK-BR-3, HCC1954, MDA-MB-468, MDA-MB-231, and BT-549 were obtained from the ATCC (Manassas, VA, USA). MCF7, MDA-MB-361, SK-BR-3, and MDA-MB-231 cells were cultured in Dulbecco modified Eagle medium (Lonza, Basel, Switzerland), and T-47D, HCC1954, MDA-MB-468, and BT-549 were maintained in Roswell Park Memorial Institute-1640 medium (Lonza); all media were supplemented with 10% fetal bovine serum (Sigma-Aldrich, St. Louis, MO, USA), penicillin (100 units/mL), and streptomycin (100 mg/mL). All cells were cultured at 37 °C in a humidified atmosphere of 5% carbon dioxide.

### Antibodies and reagents

Antibodies against IκBα (#9242), phospho-SAPK/JNK (Thr183/Tyr185; #9251), SAPK/JNK (#9252), phospho-p38 MAPK (Thr180/Tyr182; #9211), p38 MAPK (D13E1) XP rabbit (#8690), PARP (46D11) rabbit (#9532), Caspase 3 (#9662), Caspase 7 (D2Q3L; #12827), LC3A/B (D344C) XP rabbit (#12741), anti-mouse IgG (#7076), and anti-rabbit IgG (#7074) were purchased from Cell Signaling Technology (Danvers, MA, USA). Dox (#D1515) and antibody against β-actin (#A2228) were obtained from Sigma-Aldrich. Antibody against MKP-1 (M-18) was purchased from Santa Cruz Biotechnology (#sc-1102, Dallas, TX, USA). MLN9708 (#A4007) was purchased from ApexBio (Houston, TX, USA) and was prepared according to the manufacturer’s recommendations.

### Cytotoxicity assay

Cell cytotoxicity assays were performed using CCK-8 (Dojindo Molecular Technologies, Rockville, MD, USA) following the manufacturer’s instructions. Briefly, cells were seeded in 96-well plates (5 × 10^3^ cells per well). After 24 h of incubation at 37 °C, cells were either allowed to grow in medium alone (control) or in medium containing increasing concentrations of MLN9708, Dox, or a combination of the two agents. After 48 h or 72 h, cells were observed and photographed *via* optical microscope (magnification, ×200). CCK-8 (10 μL) was added to each well, and the cells were incubated for 2 h. The absorbance of each well was measured at 450 nm, and the data were plotted for the cell viability curve. Each experiment was performed in triplicate.

### Colony formation assay

Cells were seeded in 6-well plates (5 × 10^3^ cells per well). After 48 h, cells were incubated with medium alone or with MLN9708 at 0.1 μM or 0.3 μM for 72 h and then were cultured in drug-free medium for 2 weeks. Then cells were fixed and stained with methanol/crystal violet for 10 min and were photographed. Each experiment was performed in triplicate.

### Anchorage-independent growth assay

Cell anchorage-independent growth ability was assessed by soft agar assay. In 6-well plates, the bottom layer in each well was composed of semi-solid 0.5% agar (2 mL). For the top layer, cells were mixed with 0.3% agar (1.5 mL) in each well at a density of 5 × 10^3^ cells per well and were mixed with MLN9708 (0.1 μM or 0.3 μM). Untreated cells were used as controls. Cells grew at 37 °C for 3 weeks until the colonies were visible to the naked eye. Colonies were then stained with crystal violet for 2 h and were photographed. The colonies were counted by Quantity One software (Bio-Rad Laboratories, Hercules, CA, USA), and the data were plotted. Each experiment was performed in triplicate.

### Immunoblotting

For immunoblotting, after each treatment, cells were washed twice with ice-cold phosphate-buffered saline solution and were spun down. The cell pellets were dissolved in lysis buffer for 30 min (Tris-HCl [pH 7.4, 50 mM]; sodium chloride [150 mM]; EDTA [1 mM]; 1% NP-40; 0.25% sodium deoxycholate; phenylmethylsulfonyl fluoride [1 mM]; benzamidine [1 mM]; leupeptin [10 μg/mL]; dithiothreitol [1 mM]; sodium fluoride [10 mM]; sodium orthovanadate [0.1 mM]; and phosphatase inhibitor cocktail 2 and 3 [p5726 and p0044, Sigma-Aldrich]). The solutions were centrifuged at 13,000 rpm for 15 min, and the supernatants were collected as cell lysates. The cell lysates were subjected to 10% or 15% SDS-PAGE and were transferred to polyvinylidene fluoride membranes. The samples were subjected to immunoblotting with primary antibodies and with horseradish peroxidase–conjugated antibodies against rabbit or mouse IgG. The membranes were developed using the ECL Western blotting system (Thermo Fisher Scientific, Waltham, MA, USA) according to the manufacturer’s instructions.

### Statistical analysis

Statistical analysis was performed using GraphPad Prism 5 software (La Jolla, CA, USA). All values are presented as means ± standard deviation. *P* values less than 0.05, 0.01, or 0.001 were considered to be statistically significant. The Student *t*-test (two-tailed) or analysis of variance (the Dunnett multiple comparison post-test) was used to analyze the difference between the drug treatment groups and the control group.

## Additional Information

**How to cite this article**: Wang, H. *et al*. Next-generation proteasome inhibitor MLN9708 sensitizes breast cancer cells to doxorubicin-induced apoptosis. *Sci. Rep*. **6**, 26456; doi: 10.1038/srep26456 (2016).

## Figures and Tables

**Table 1:** Molecular classification of human breast cancer cell lines^26^,^27^,^28^.

Cell line	Subtype	ER*	PR*	HER2*	Source	Tumor type
T-47D	LuA	+	[+]		PE	IDC
MCF7	LuA	+	[+]		PE	IDC
MDA-MB-361	LuB	+	[−]	+	P.Br	AC
SK-BR-3	HER2	−	[−]	+	PE	AC
HCC1954	HER2	−	[−]	+	P.Br	Duc.Ca
MDA-MB-468	BaA	[−]	[−]		PE	AC
MDA-MB-231	BaB	−	[−]		PE	AC
BT-549	BaB	−	[−]		P.Br	IDC, pap

Abbreviations: ER, estrogen receptor; PR, progesterone receptor; HER2, human epidermal growth factor receptor 2; AC, adenocarcinoma; BaA, basal A; BaB, basal B; Duc.Ca, ductal carcinoma; IDC, invasive ductal carcinoma; Lu, luminal; Pap, papillary; P.Br, primary breast; PE, pleural effusion.

^*^ER/PR/HER2 status: ER/PR positivity and HER2 overexpression (obtained from the Sanger website as indicated). Square brackets indicate that levels were inferred from mRNA levels alone when protein data were unavailable.
